# The Genus *Cordyceps* Sensu Lato: Their Chemical Constituents, Biological Activities, and Therapeutic Effects on Air Pollutants Related to Lung and Vascular Diseases

**DOI:** 10.3390/life15060935

**Published:** 2025-06-10

**Authors:** Hye-Jin Park

**Affiliations:** Department of Veterinary Medicine, College of Veterinary Medicine, Konkuk University, Seoul 05029, Republic of Korea; nimpi79@hanmail.net

**Keywords:** *Cordyceps*, environmental lung disease, biological activities, traditional Chinese medicine

## Abstract

Air pollutants are significant environmental factors that contribute to the exacerbation of respiratory, cardiopulmonary, and skin diseases in East Asia, and their impact is based on particle size. Natural products represent a promising and sustainable strategy for reducing the adverse effects of air pollutants on health. *Cordyceps* spp. have been integral to traditional Chinese medicine. Recently, their fruiting bodies and related supplements have gained popularity. The physiological effects of *Cordyceps* species are well documented and attributed to their chemical constituents, such as cordycepin, polysaccharides, cordymin, glycoprotein, ergosterol, and other bioactive extracts. *Cordyceps* supplementation may support lung health and enhance respiratory function. Although further clinical data are necessary, many preclinical studies have found a connection between *Cordyceps* and improved lung health. In addition, preclinical and clinical studies have indicated that *Cordyceps* and its derivatives (e.g., Ningxinbao, Corbrin, and Jinshuibao capsules) protect against vascular diseases by modulating key molecular pathways. This review provides insights into the potential of *Cordyceps* for clinical application in the management of air pollutant-related respiratory and vascular diseases.

## 1. Introduction

Exposure to air pollutants is associated with numerous health issues, including unexpected death among individuals with vascular disorders, asthma, irregular heartbeat, heart attack, lung disease, and reduced lung function [1]. Naturopathic medicines derived from natural sources, including microorganisms, plants, animals, and marine organisms, are recognized for their beneficial effects in the prevention and treatment of various diseases. Naturopathic remedies typically have fewer side effects than allopathic medicines. Traditionally, these remedies have been utilized worldwide in minimally invasive yet effective ways and are rich in antioxidants and anti-inflammatory compounds, which may counteract the adverse effects of particulate matter (PM) on the human body. Medicinal mushrooms have been used in naturopathic medicine to prevent and treat various diseases. Among them, the genus *Cordyceps*, which comprises approximately 750 varieties and a group of ascomycete parasitic fungi, is primarily used as a functional ingredient in traditional Oriental medicine in Asia [2,3]. *Cordyceps militaris* (*C. militaris*) has been reported to have immunoprotective, antimicrobial, antiviral, anticancer, anti-inflammatory, and antioxidant properties [4]. Consequently, it has gained significant attention as a potential novel agent for combating respiratory and vascular diseases related to exposure to air pollutants. Recent advances in fungal taxonomy based on molecular phylogenetic analyses have led to the reclassification of the genus *Cordyceps*, with several species reassigned to new genera, such as *Ophiocordyceps* and *Metacordyceps*. Despite these revisions, the term *Cordyceps* remains widely used in the pharmacological, clinical, and ethnobotanical literature to collectively describe the traditional group of entomopathogenic fungi with medicinal value. In this study, we adopted the classical concept of *Cordyceps* (*sensu lato*, abbreviated as s.l.) to include all conventionally recognized medicinal species, both formerly and currently classified under the genus *Cordyceps* [5,6]. This approach ensures continuity with the existing literature and supports its widespread relevance in therapeutic research and development.

## 2. Genus *Cordyceps*: Promising Health Benefits for Managing Various Disease Conditions

**Figure 1:**
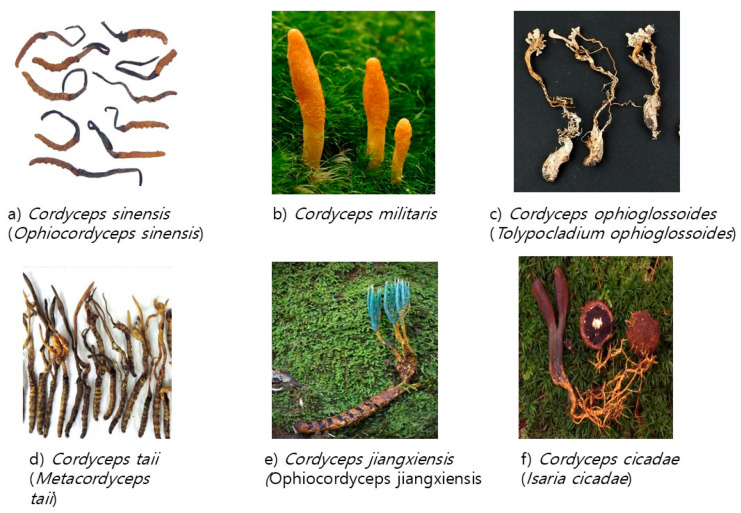
Images of *Cordyceps* species (**a**) *Cordyceps sinensis* (*Ophiocordyceps sinensis*); (**b**) *Cordyceps militaris*; (**c**) *Cordyceps ophioglossoides* (*Tolypocladium ophioglossoides*); (**d**) *Cordyceps taii* (*Metacordyceps taii*); (**e**) *Cordyceps jiangxiensis* (*Ophiocordyceps jiangxiensis*); (**f**) *Cordyceps cicadae* (*Isaria cicadae*). The images are sourced from MeFSAT [13] and Wikimedia Commons in the public domain.

**Figure 2:**
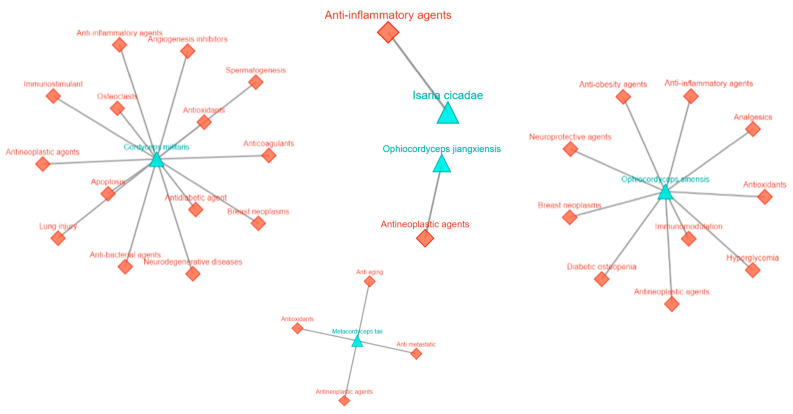
Health benefits of the genus *Cordyceps* in managing various disease conditions. Images modified from MeFSAT (https://cb.imsc.res.in/mefsat/ accessed on 22 March 2025).

Historically, the term “*Cordyceps*” has primarily referred to *C. sinensis*. The two most extensively studied species of the *Cordyceps* genus are *C. sinensis* and *C. militaris*. *Cordyceps* species exhibit various biological functions, showing potential for treating respiratory and liver dysfunctions, heart diseases, cardiovascular diseases, uncontrolled perspiration, fatigue, impotence, spermatorrhea, and cancers [7] (Figure 1 and Figure 2). The State Food and Drug Administration of China has approved 50 medicines and 2 dietary supplements containing *Cordyceps militaris* and *Cordyceps sinensis* [8]. The market efficacy of these two *Cordyceps* species has significantly increased since the 1980s. In 2023, the global market for *C*. *militaris* was estimated to be worth USD 1.02 billion. Its annual growth rate (CAGR) of 11.79% from 2023 to 2033 is expected to result in significant expansion. By 2033, the global market for *C. militaris* is projected to reach USD 3.11 billion [9]. This demand has resulted in the overharvesting of *C. sinensis*, resulting in its near-total depletion. Due to their rarity and high cost, most wild *Cordyceps* species are now artificially cultivated. Numerous studies on artificially cultured species have shown promising results, indicating that these cultivated species possess the same potent bioactivities as their wild counterparts [3]. Owing to the limited presence of *C. militaris* in natural habitats, we opted to cultivate it on germinated *Rhynchosia nulubilis* (GRC) [3]. Notably, when cultivated on germinated soybeans (GSC), *C. militaris* exhibits immunostimulatory [10], anticancer [11], and antiallergic properties [12].

### 2.1. Cordyceps sinensis

*C. sinensis*, now renamed as “*Ophiocordyceps sinensis*”, is one of the most widely studied species. It has been used for a long time in traditional Chinese medicine (TCM). The consumption of *C. sinensis* is recommended for preventing infections and flu because of its efficacy in reducing phlegm, cough, and bronchial disease symptoms [11]. The *C. sinensis* capsule significantly reduced the frequency and severity of acute exacerbations in patients with chronic bronchitis compared to the placebo. It also improved symptoms such as expectoration and wheezing, with no significant difference in adverse event rates between the groups [14]. Adjuvant *Cordyceps sinensis* treatment in patients with lung cancer improves tumor response, quality of life, and immunity while reducing adverse reactions and radiation pneumonitis [15]. Cordymin, a peptide purified from *C. sinensis*, has anti-inflammatory and antinociceptive effects [16]. Consequently, *Cordyceps* spp. have been used to treat lung fibrosis, particularly in patients with severe acute respiratory syndrome (SARS). These therapeutic effects align with TCM principles, attributing them to the capacity of *C. sinensis* to balance lung yin and yang [17]. In addition, *C. sinensis* enhances cardiac energy metabolism, prevents calcium overload during myocardial ischemia, and alleviates blood–brain barrier disruption induced by cerebral ischemia [18]. It also helps prevent or treat hypertension, thrombosis, atherosclerosis, and arrhythmia linked to heart ischemia. Fermented *C. sinensis* powder reduces inflammation and pulmonary arteriole remodeling in hypoxia-induced pulmonary hypertension in rats by blocking the p38 MAPK and NF-κB signaling pathways [14].

### 2.2. Cordyceps militaris

*C. militaris*, which is often cultivated and used in TCM, shares several medicinal qualities with *C. sinensis*. *C. militaris* is used to treat a spectrum of conditions, including pulmonary and kidney impairment, elevated blood sugar levels, abnormal lipid profiles, breathing difficulties, exhaustion, night sweats, reproductive issues, irregular heart rhythms, and other heart ailments. Its pharmacological properties include anti-inflammatory, antioxidant, anticancer, antimetastatic, immunomodulatory, hypoglycemic, and steroidogenic activities [19]. CM1, a purified polysaccharide from *C. militaris*, significantly reduced atherosclerotic plaque formation and improved lipid profiles in LDLR (−/−) mice. It regulates lipid metabolism through multiple pathways, including enhancing VLDLR expression, suppressing hepatic lipid synthesis, and promoting intestinal lipid excretion [20]. Hyperlipidemia, a key cardiovascular risk factor, is often treated with synthetic drugs that have adverse effects. Cordycepin from *C. militaris* reduces triglycerides, cholesterol, LDL, and VLDL in hyperlipidemic rats by activating AMPK and inhibiting hepatic lipase and lipoproteins, offering natural therapeutic potential [19]. *C. militaris* cocktail inhibits inflammation and fibrosis in lung tissue caused by PM [21]. *Cordyceps militaris* ARA301 extract (CME) significantly attenuated lipopolysaccharide (LPS)-induced lung injury in mice by reducing immune cell infiltration, mucus production, and pro-inflammatory cytokine levels. It also suppressed NF-κB pathway activation in vitro, suggesting its potential as a functional supplement for lung protection [22]. A functional beverage containing fermented *Cordyceps militaris* (FCM) enhanced NK cell activity and reduced pro-inflammatory cytokines (IL-1β, IL-6) in healthy men and women without adverse effects. These studies indicate that FCM may be a safe and effective immune-enhancing supplement [23].

### 2.3. Other Cordyceps Species

Mushrooms grown on insect substrates, such as *C. fumosorosea*, are considered promising agents for managing various insect populations through biological control. Fumosoroseanosides A and B from *C. fumosorosea* exhibit anti-aging, antibacterial, and antifungal activities [24]. Serum metabolomic analysis in mice revealed the alleviating effect of *C. fumosorosea* against acute lung injury (ALI) [25].

*Metacordyceps taii* develops on the larvae or pupae of *Hepialidae* that reside in the soil or in tree trunks [26]. The chloroform extract of *Metacordyceps taii* (CFCT) demonstrated in vivo antitumor and antimetastatic activities, indicating its potential for development as a chemopreventive agent derived from *Metacordyceps taii* [27]. *Metacordyceps taii* polysaccharides demonstrated strong immune function-boosting, antioxidant effects, and free radical scavenging activity [28].

*C. jiangxiensis* thrives in moist soil, particularly on the larvae of Elateridae (Coleoptera) [26]. A novel compound, jiangxienone, isolated from *C. jiangxiensis*, exhibits strong cytotoxic effects on human lung carcinoma (A549) and human gastric adenocarcinoma (SGC-7901) cells [29].

*Isaria cicadae* may ameliorate kidney fibrosis by targeting the TGF-β1 and CTGF pathways in vivo [30]. Aqueous extracts of *Isaria cicadae* (*Miq*.) Massee have been reported to be a promising hydrating and antiwrinkle component that enhances hyaluronan concentration in human skin fibroblasts [31]. This species could reduce inflammation, oxidative stress, and renal fibrosis in the murine model with lupus nephritis by modulating the PI3K/mTOR-mediated autophagy pathway [31]. *Isaria cicadae* mycelium (CCM) ameliorates high intraocular pressure in animals and humans [32]. It also prevents early-stage cataracts in a UVB-induced murine cataract model [32].

*C. ophioglossoides* exhibits antitumor activity that is attributed to an alkali-soluble polysaccharide present in the fungus [33]. Ophiocordin isolated from *C. ophioglossoides* showed antifungal antibiotic effects [34].

### 2.4. Bioactive Constituents of Cordyceps Species

In addition to the major bioactive compounds described above, recent studies have identified over 100 distinct chemical constituents in various *Cordyceps* species. These include nucleosides (e.g., adenosine, cordycepin), polysaccharides, sterols (e.g., ergosterol, β-sitosterol), peptides, alkaloids, flavonoids, and cyclic dipeptides [2,4].

More than 30 compounds have been isolated from *Cordyceps militaris*, and over 70 compounds, including unique fatty acids and complex protein-bound polysaccharides, have been identified in *C. sinensis* [4].

This wide range of chemical constituents highlights the metabolic richness of individual *Cordyceps* species and supports their diverse pharmacological activities.

## 3. Fermented *Cordyceps* and Its Role in Immune Regulation: Evidence from Recent Studies

*C. militaris* is a fungal species that grows by parasitizing insect larvae. However, the production yield of *C. militaris* in nature is very low because of the requirement for specific hosts and stringent growth conditions. The commercial use of natural *C. militaris* is limited by its high cost and low extraction yield [3,35,36]. Cari Co., Ltd. grew *C. militaris* on germinated *R. nulubilis* (GRC) instead of using dead insects [3]. GRC fermented with *Pediococcus pentosaceus* SC11, a strain isolated from salted small octopus, possesses immune-enhancing and antiviral properties. The lactic acid bacteria fermentation process with SC11 significantly increased the contents of total flavonoids, cordycepin, and β-glucan in GRC. Notably, GRC-SC11 suppresses 3CL protease activity associated with severe acute respiratory syndrome coronavirus (SARS-CoV). Furthermore, studies involving immunocompromised mice have shown that GRC-SC11 significantly enhances the indices of the thymus and spleen [3]. Treatment with *C. militaris*, fermented by *P. pentosaceus* ON89A, a strain isolated from onion (GRC-ON89A), has been shown to restore immune function in mice treated with high doses of cyclophosphamide, a chemotherapeutic drug. This recovery is evidenced by increased phagocytic activity and nitric oxide (NO) production in mouse peritoneal macrophages. Additionally, GRC-ON89A mitigates the toxicity of anticancer agents by aiding immune system restoration [37]. Fermented *C. militaris* with a *P. pentosaceus*, a strain isolated from a small salted octopus (SC11), significantly reduced IgE-induced allergic reactions in BALB/c mice with passive cutaneous anaphylaxis (PCA) by decreasing inflammatory cell infiltration, vascular permeability, and ear swelling. This fermentation process enhanced the antiallergic effects of GRC, boosting both cordycepin content and antioxidant activity compared to non-fermented GRC [36]. Enzymatic breakdown and fungal fermentation significantly enhanced the flavor and nutritional value of *C. militaris* beverages. Enzymatic breakdown generates a more reduced form of sugar, providing an optimal fermentation medium for bacterial growth. A previous study revealed that using a mixture of cellulase and pectinase at a 2:3 ratio resulted in elevated cordycepin levels. After fermentation, the cordycepin concentration remained high at 98.02% [38].

## 4. *Cordyceps* Species and Their Bioactive Substances Effective for Air Pollutant-Induced Respiratory Diseases

The lungs are strongly influenced by environmental factors [39]. Air pollutant exposure significantly increases the incidence of respiratory diseases, with indoor and outdoor air pollution responsible for seven million fatalities worldwide [40]. Air pollution is caused by various chemical and biological elements present in both indoor and outdoor environments. Harmful outdoor air pollutants for the respiratory system include gas chemicals such as ozone (O_3_), nitrogen oxide including NO_2_, sulfur dioxide (SO_2_), inhaled diesel gas and PM, and allergens in the air, such as endotoxin (e.g., lipopolysaccharide), fungal spores, allergenic, and pollen [41]. The inhalation of particulate matter (PM) in ambient air increases the risk of acute lower respiratory infections by bacteria and viruses, pneumonia, and asthma in children [41,42]. Outdoor pollutants can also affect indoor air quality. Indoor air contains various harmful substances from numerous sources. In indoor environments, one can find environmental tobacco smoke (ETS), NO_2_, formaldehyde (HCHO), other volatile organic compounds (SO_2_, O_3_), and allergens derived from various plants, animals, and insects [43]. Exposure to indoor cigarette smoke increases the incidence of pediatric respiratory diseases, including asthma, rhinitis, and respiratory tract infections [43]. *Cordyceps*, a genus of parasitic fungi, has been used in traditional Oriental medicine for its potential health benefits in treating various lung disorders, including chronic obstructive pulmonary disease (COPD). This study provides an overview of its relevance and potential benefits in the management of lung conditions (Table 1).

### 4.1. COPD Treatment

Epidemiological and mechanistic studies have shown a close association between air pollution and the development of COPD. The primary cause of COPD is long-term exposure to harmful substances that damage the lungs, most commonly cigarette smoke. Other causes include air pollution, occupational dust, chemicals, and secondhand smoke [63]. Studies have shown that *Cordyceps* may improve lung function in patients diagnosed with GOLD stages 2 and 3 COPD. A systematic review and meta-analysis found that the combination of *Cordyceps* and Western medicine significantly improved lung function metrics, such as the volume exhaled at the end of the first second of forced expiration (FEV1)% predicted and the FEV1/forced vital capacity (FVC) ratio, which are critical measures of pulmonary function in patients with COPD [64]. *C. sinensis* treatment led to a significant reduction in airway wall thickening, including collagen deposition, smooth muscle hypertrophy, fibrosis, and epithelial hyperplasia in a COPD rat model [65]. Nucleosides isolated from *C. sinensis* alleviate cigarette smoke extract-triggered inflammation through the SIRT1-NF-κB/p65 pathway in RAW264.7 macrophages and mice with COPD. *Cordyceps*-derived nucleosides and ergosterol have demonstrated therapeutic effects in a COPD model. Nucleosides activate SIRT1 and suppress the NF-κB/p65 pathway while also reducing TGF-β1/Smad signaling and increasing Smad7 expression, thereby attenuating airway inflammation and fibrosis in RAW264.7 macrophages and mice with COPD [66]. Ergosterol suppresses cigarette smoke extract-induced COPD by mitigating inflammation, oxidative stress, and apoptosis both in vitro and in vivo [56]. Recent studies have demonstrated that *Cordyceps militaris* cultivated on germinated *Rhynchosia nulubilis* and encapsulated in chitosan nanoparticles (GCNs) significantly enhances the expression of superoxide dismutase 1 (SOD1) in the lung tissues of mice exposed to PM2.5-induced oxidative stress [67]. SOD1, a Cu-Zn-dependent cytosolic antioxidant enzyme, plays a crucial role in neutralizing reactive oxygen species (ROS) and protecting lung epithelial integrity. The recombinant Cu, Zn-SOD from *Cordyceps militaris* retained 80 ± 2% activity under physiological conditions and showed structural similarity to the native enzyme, suggesting stable antioxidant capacity. Given the role of oxidative stress in the pathogenesis of COPD, its functional resemblance and stability support the therapeutic potential of *C. militaris*-derived SOD in mitigating pulmonary oxidative damage [68]. Cordycepin from *Cordyceps militaris* exerts immunomodulatory effects by suppressing T cell activation through the inhibition of the TCR signaling cascade, as demonstrated in CFA-induced inflammation models. This mechanism may contribute to its therapeutic potential in COPD, where dysregulated T cell responses and chronic inflammation play a critical role [69]. The hot-water extract from *C. guangdongensis* reduced tobacco smoking-induced chronic bronchitis symptoms [44].

### 4.2. Anti-Asthma

Air pollutants, such as PM2.5 and formaldehyde (FA), exacerbate allergic asthma through oxidative stress. Asthma is a clinical condition characterized by intermittent respiratory symptoms, typically marked by generalized airway hyperreactivity and inflammation. *C. sinensis* and *C. militaris* were studied for their effects on pulmonary function using a Calu-3 cell line, a human airway epithelial model. Both extracts, along with their active compounds, cordycepin and adenosine, enhanced ion transport in a dose-dependent manner. The study found that these extracts influence anion movement in airway epithelia, involving the basolateral Na^+^–K^+^–2Cl^−^ symporter and the apical cAMP-regulated CFTR Cl^−^ channel. The effects of *C. sinensis* and *C. militaris* on anion movement contribute to the hydration of mucus in the airways, leading to a low-viscosity mucus layer. This reduced viscosity is expected to improve mucociliary clearance, which may be beneficial for treating certain pulmonary diseases such as asthma [70]. Cordycepin from *C. militaris* inhibits NF-κB pathway, suppresses Th2 cytokines (IL-4, IL-5, IL-13), reduces eosinophilic inflammation, and improves airway hyperresponsiveness [71]. *Cordyceps* polysaccharides regulate Th1/Th2 balance, reduce IgE levels, and inhibit mast cell degranulation [72]. *Cordyceps militaris* polysaccharide (CMP) alleviated allergic asthma in ovalbumin-induced mice by reducing lung and gut inflammation, improving oxidative stress, and modulating Nrf2/HO-1 and NF-κB signaling pathways. CMP also restored gut microbiota composition and function, suggesting gut–lung axis involvement in its therapeutic effects [73].

### 4.3. Antiviral Activity

Exposure to environmental PM may be associated with increased vulnerability to viral infections [74]. It is reported that *Cordyceps* exerts antiviral activities owing to the presence of active compounds. Cordycepin, currently being tested in clinical trial NCT00709215, shares structural resemblance with adenosine but lacks a 3′ hydroxyl group in its ribose structure. This difference allows cordycepin to act as a poly(A) polymerase inhibitor, leading to the premature termination of protein synthesis. Given that functional RNAs of the SARS-CoV-2 genome are highly 3′-polyadenylated, cordycepin interferes with the synthesis of all viral proteins [45]. Steroids extracted from *C. militaris* have been recognized as crucial for controlling the cytokine storm associated with COVID-19 [46]. *Cordyceps* appears to be a safe immune booster for the treatment of patients with mild-to-moderate COVID-19 [75]. Ohta et al. investigated the anti-influenza effects of *C. militaris* and observed a significant reduction in virus titers in both the lung tissue and bronchoalveolar lavage fluid of mice administered intranasally with an acidic polysaccharide (APS) extracted from *C. militaris* [47]. *C. guangdongensis* exerted anti-influenza viral activity [44]. The *C. militaris* group exhibited notably higher NK cell activity (*p* = 0.047) and IgA levels (*p* = 0.035) than the placebo group [76]. *C. militaris* powder could potentially reduce lung inflammation and fibrosis triggered by the SARS-CoV-2 spike protein and LPS through the TGF-β R1/Smad2 signaling pathway [77].

### 4.4. Lung Cancer Treatment

Air pollution is the second most common cause of lung cancer. When combined with smoking, it has a synergistic effect that worsens lung cancer survival [78]. In studies on non-small cell lung cancer (NSCLC), *C. sinensis* has demonstrated potential anticancer properties. It has been reported to inhibit tumor growth and metastasis by affecting pathways, such as the MAPK pathway, which is involved in cell proliferation and survival (BioMed Central). Several studies have demonstrated that cordycepin (3′-deoxyadenosine), a key bioactive compound extracted from *C. militaris*, inhibits cancer cell proliferation [58,79]. Cordycepin blocks the growth of Lewis lung carcinoma cells in vitro by activating adenosine A3 receptors [58]. Cordycepin triggers apoptosis in human lung cancer cells by blocking the nitric oxide-stimulated ERK/Slug signaling pathway [50]. Cordycepin treatment increases apoptosis in human lung adenocarcinoma by upregulating Foxo3a via caveolin-1-mediated JNK signaling [51]. Cordycepin reduced the viability of A549 and PC9 human lung adenocarcinoma cells by suppressing LPS-induced expression of iNOS, NO, phospho-ERK (p-ERK), and Slug. It induced apoptosis by inhibiting the ERK/Slug pathway via GSK3β activation, leading to increased Bax and caspase-3 expression in lung cancer cells [50]. *C. sinensis* ameliorates non-small cell lung cancer by blocking the MAPK pathway [80]. *C. sinensis* aqueous extract enhances the antitumor efficacy of cisplatin while mitigating therapy-related toxic effects against non-small cell lung cancer through AKT/MMP2/MMP9 and NFkB pathways [81]. *C. sinensis* extract suppresses breast cancer cell metastasis by inhibiting the expression of metastasis-related cytokines [82]. A polysaccharide-rich extract from *Cordyceps sinensis* (CS) significantly enhanced the cytotoxic and pro-apoptotic effects of cisplatin in H157 non-small cell lung cancer (NSCLC) cells. The combination treatment also reduced VEGF and bFGF expression, suggesting that CS may serve as a potential adjuvant in NSCLC chemotherapy [83]. Cordyceps acid from *C. sinensis* exhibits a significant tumor-suppressing effect on lung cancer by modulating the Nrf-2/HO-1/NLRP3/NF-κB signaling pathway [60]. *C. sinensis* (hot-water extract) inhibits B16 melanoma cell lung metastasis [84].

### 4.5. Acute Lung Injury Treatment

Chronic (rather than short-term) exposure to low-to-moderate levels of air pollutants, such as O_3_, NO_2_, SO_2_, carbon monoxide (CO), and PM ≤ 2.5 μm in aerodynamic diameter (PM2.5), is linked to the onset of acute lung injury (ALI) [85]. *C. sinensis* and its active compound cordycepin could have an anti-inflammatory and antioxidant effect on LPS-induced ALI by suppressing NF-κB p65 phosphorylation, as well as the expression of COX-2 and iNOS in the lungs [61]. Cordycepin, a natural compound derived from *C. militaris*, was found to reduce lung edema, the production of inflammatory cytokines (TNF-α, IL-1β, and IL-6) and nitric oxide, MPO activity, and malondialdehyde (MDA) content in LPS-induced ALI mice [48,86]. Studies have examined the ability of *C. fumosorosea* to alleviate ALI. Metabolomic analysis in mice indicated that this species could reduce lung inflammation and improve overall lung health by altering various metabolic pathways and reducing oxidative stress [25]. According to the Medicinal Fungi Secondary Metabolite And Therapeutics (MeFSAT) database, miR-1321 and miR-3188, both present in *C. militaris*, target the 3′-UTR of CXCR2 mRNA, thereby inhibiting its translation in the lungs of bleomycin-treated mice [52]. Cordycepin treatment significantly inhibited the increase in the lung wet/dry weight ratio, MPO activity, MDA content, and inflammatory cytokine production in LPS-induced ALI mice. Moreover, cordycepin inhibited LPS-induced NF-κB activation and increased Nrf2 and HO-1 protein expression in a dose-dependent manner [52]. Treatment with cigarette smoke extract (CSE) increased cellular senescence and activated the ROS/PI3K/AKT/mTOR pathway in human bronchial epithelial (16HBE) cells, both of which were reduced by *C. sinensis*. Blocking this signaling pathway can reduce CSE-induced cellular senescence [57]. *Cordyceps militaris*-derived polysaccharides significantly alleviated LPS-induced ALI in mice by reducing inflammatory cytokines (e.g., TNF-α, IL-6) and oxidative stress markers. These effects are mediated by the suppression of NF-κB activation and enhancement of endogenous antioxidant defenses [87]. *Cordyceps militaris* (GCN) encapsulated in chitosan nanoparticles significantly alleviated PM2.5-induced lung injury in mice by reducing oxidative stress, suppressing inflammation, and restoring epithelial barrier integrity. GCN also enhanced antioxidant enzyme expression and inhibited ECM degradation, showing potential as a therapeutic agent for pollution-related lung diseases [88].

### 4.6. Idiopathic Pulmonary Fibrosis (IPF) Treatment

Numerous studies support a causal link between air pollution and idiopathic pulmonary fibrosis (IPF) [89]. Idiopathic pulmonary fibrosis is a chronic, irreversible, and debilitating lung disease characterized by the expansion of fibroblasts and myofibroblasts and abnormal buildup of the extracellular matrix in the interstitium, resulting in breathing difficulties [54]. A combined pharmacokinetic and pharmacological analysis revealed that *Cordyceps* extract and its active components, such as cordycepin and adenosine, exhibit antifibrogenic properties by inhibiting epithelial–mesenchymal transition (EMT) [54]. The anamorph of *C. sinensis* attenuates bleomycin-induced pulmonary inflammation and fibrosis in vivo [90]. The phenotypic transition of cobblestone-shaped epithelial cells into more mobile myofibroblasts, known as EMT, along with the formation of fibroblastic foci, are prominent characteristics of both experimental and human lung fibrosis, such as IPF [54].

*C. sinensis* enhances hypoxia tolerance in human lung epithelial cells by increasing the expression of Heme Oxygenase-1 and metallothionein via Nrf2 activation in human lung epithelial cells [91]. *Cordyceps sinensis* ameliorates BLM-induced pulmonary fibrosis by regulating mitochondrial oxidative phosphorylation and inhibiting mitochondrial ROS overproduction. This protective mechanism involves the modulation of mitochondrial complex expression and activity, thereby preserving mitochondrial integrity and function [92].

Ko’s group reported that *C. sinensis* and *C. militaris* enhance respiratory health by modulating the activity of the Na^+^–K^+^–2Cl^−^ cotransporter and the CFTR Cl^−^ channel. These mushrooms improve ion transport and fluid balance in the airway epithelium, offering potential therapeutic benefits for respiratory diseases [70].

Extracts of *Cordyceps militaris* containing cordycepic acid (D-mannitol) have been suggested to alleviate mucus hypersecretion by modulating several pathological mechanisms associated with airway inflammation and epithelial dysfunction. Although the direct effect of cordycepic acid on mucus production remains unclear, studies have shown that *C. militaris* extracts can reduce airway goblet cell hyperplasia and mucus-related gene expression in respiratory inflammation models, indicating their potential indirect role in regulating mucus overproduction [93,94].

### 4.7. Silicosis Treatment

Silicosis is a fibrotic lung disorder caused by occupational and air pollutant exposure and is prevalent worldwide, particularly in developing countries. Fermented *C. sinensis* may alleviate silica-triggered pulmonary inflammation and fibrosis by suppressing Th1 and Th17 immune responses and preventing the augmentation of Th2 responses [49]. Fermented *C. sinensis* powder (FCP) mitigates silica-induced pulmonary fibrosis by modulating immune responses. Specifically, FCP suppresses overactive Th1 and Th17 responses and inhibits the enhancement of Th2 responses, thereby reducing inflammation and fibrotic progression in the lungs [49].

## 5. *Cordyceps* Undergoing Clinical Trials

Enhanced NK cell activity was observed in healthy volunteers who were administered *C. militaris* capsules containing 7.3% cordycepic acid, 0.13% adenosine, 0.001% cordycepin, and 32% cordyceps polysaccharide, indicating that *C. militaris* could potentially serve as an immune-stimulating agent [23] (Table 2).

*Cordyceps sinensis* (Cs-4) improves lung function and enhances exercise performance in healthy older adults. After 12 weeks of Cs-4 supplementation, the metabolic threshold, where lactate begins to accumulate, increased by 10.5%, from 0.83 ± 0.06 to 0.93 ± 0.08 L/min (*p* < 0.02). Similarly, the ventilatory threshold, which reflects the point where unbuffered H+ stimulates ventilation, increased by 8.5%, from 1.25 ± 0.11 to 1.36 ± 0.15 L/min. These findings suggest that Cs-4 can enhance lung function by improving oxygen uptake, aerobic capacity, ventilatory efficiency, and fatigue resistance in older adults [98]. The administration of Bailing capsules (Cs-C-Q80) in patients with chronic bronchitis significantly reduced the frequency of acute exacerbations and improved key respiratory symptoms, such as expectoration and wheezing. The treatment was well tolerated, with no significant difference in adverse events compared to those in the placebo group. These findings suggest that Bailing capsules may be an effective and safe adjunct therapy for the management of chronic bronchitis [14]. The fruiting bodies of *C. militaris* have shown the potential to alleviate urinary symptoms and decrease the size of the prostate gland [95]. *C. militaris* extract is expected to be safely used as a functional food to protect against the progression of fatty liver or cirrhosis by suppressing the lipid accumulation of hepatocytes in patients with mild liver dysfunction [96]. Adjuvant treatment with CS for lung cancer not only improves the tumor response rate, quality of life, and immune function but also decreases the occurrence of adverse drug reactions (ADRs) and radiation pneumonitis [15]. *Paecilomyces hepiali* (CBG-CS-2) contains *Cordyceps* polysaccharides and adenosine, which play a crucial role in initiating immune responses and inducing immunomodulatory effects. This is achieved by enhancing NK cell activity and phagocyte reactions through activation [101].

Despite increasing clinical interest, the standardization of *Cordyceps* spp. formulations remains a challenge due to variability in strains, cultivation methods, and extraction techniques. To ensure reproducibility and clinical translation, future studies should report the exact content of bioactive compounds (e.g., cordycepin, adenosine, and polysaccharides) and adopt chemical fingerprinting and HPLC-based quantification for quality control purposes.

## 6. *Cordyceps* Species and Their Bioactive Substances Effective for Air Pollutant-Induced Vascular Diseases

Air pollutants have been linked to an increase in the incidence of various diseases, including cardiovascular and cerebrovascular diseases [102,103]. Among air pollutants, PM with a diameter of 10 μm or smaller (PM10) and PM2.5 have recently attracted significant attention due to their substantial impact on public health [104], respiratory disorders [105], and higher rates of neonatal mortality [106]. Cardiovascular and cerebrovascular diseases (CCVDs) are major global health concerns because of their high morbidity and mortality rates, primarily driven by atherosclerosis and blood clot formation. The key risk factors include PM, genetic predisposition, obesity, smoking, elevated cholesterol levels, and high blood pressure. Acute ischemic and reperfusion injuries in the brain (cerebral ischemia/reperfusion injury [CI/RI]) and heart (myocardial ischemia/reperfusion injury [MI/RI]) arise from reduced blood supply due to vascular stenosis or atherosclerosis. These injuries involve complex pathological processes, including autophagy, necrosis, apoptosis, inflammation, calcium overload, and oxidative stress. Moreover, clinical and experimental evidence has highlighted the link between brain injury and cardiac dysfunction, emphasizing the need for treatments targeting multiple injury mechanisms. Since the late 20th century, Chinese medicine has provided increasing evidence of *the potential of Cordyceps* in treating CCVDs. Preclinical and clinical studies suggest that *Cordyceps* and its artificial derivatives, such as Ningxinbao, Corbrin, and Jinshuibao capsules, offer protective benefits against ischemic conditions by addressing the molecular mechanisms involved in these diseases. *C. militaris* demonstrates strong potential as a natural antihypertensive agent by inhibiting angiotensin-converting enzyme (ACE) activity. *C. militaris* extract and its active compound, cordycepin, may act as natural ACE inhibitors with promising therapeutic potential for managing hypertension [107].

### 6.1. Antithrombosis

Particulate matter exposure leads to vascular injury, the release of adhesion molecules, platelet activation, and thrombin generation, collectively contributing to a prothrombotic state [108]. A novel serine protease, CSP, was purified from the culture supernatant of *Cordyceps* mycelia. CSP exhibits fibrinolytic activity and efficiently degrades fibrinogen, fibrin, and casein. It has the potential to be a therapeutic agent for thrombosis treatment [108]. The antithrombotic activity of the ethanol extract of cultured *C. militaris* CMEE is related to its antiplatelet rather than the anticoagulation effect in an FeCl_3_-induced arterial thrombosis model [109]. *C. sinensis* (Cs)-4 polysaccharides significantly reduced CD62P expression and αIIbβ3 activation on platelets while inhibiting collagen-induced platelet activation and aggregation [109]. WIB-801CE, a cordycepin-enriched extract from *C. militaris*, exhibited significant antiplatelet effects by inhibiting platelet aggregation induced by ADP, collagen, and thrombin. This effect was associated with reduced thromboxane A2 (TXA2) and serotonin release, achieved by suppressing cyclooxygenase-1, TXA2 synthase, and cytosolic phospholipase A2 activity. These findings indicate that WIB-801CE may help prevent thrombotic diseases by reducing platelet aggregation [110].

### 6.2. Anti-Atherosclerosis

Atherosclerosis remains a major global health challenge, prompting ongoing research on innovative treatments. Studies on hypercholesterolemic rabbits and ApoE-null mice have revealed that ambient PM exposure accelerates atherosclerosis, with smaller particles having stronger proatherogenic effects [111]. *Cordyceps*, as used in TCM, has shown potential as a therapeutic agent for atherosclerosis owing to its anti-inflammatory, antioxidant, cholesterol-lowering, and platelet aggregation-inhibiting properties, as well as its effects on apoptosis and autophagy in vascular endothelial cells [112]. Studies have emphasized the pharmacological role of adenosine and cordycepin in the treatment of atherosclerosis through their anti-inflammatory, antioxidant, hypolipidemic, immunomodulatory, antiplatelet aggregation, and vascular smooth muscle relaxation effects. *Cordyceps* contains key amino acids, including glutamic acid, arginine, and aspartic acid, and its mycelia are rich in proteins, peptides, and amino acids, such as glutamic acid, phenylalanine, and aspartic acid, which contribute to energy production and exert antithrombotic and vasodilatory effects associated with their potential to prevent and manage atherosclerosis.

### 6.3. Anticerebral Ischemic/Reperfusion Injury

Increasing evidence indicates that exposure to airborne fine PM2.5 is associated with an increased risk of ischemic stroke [113]. Cordycepin, a bioactive compound derived from *C. militaris*, has demonstrated strong neuroprotective effects in cerebral ischemia/reperfusion models. In both mice and brain slices, cordycepin reduced neuronal degeneration, reduced the levels of excitatory amino acids such as aspartate and glutamate, boosted SOD activity, and reduced MDA, thus decreasing oxidative stress. It also inhibited matrix metalloproteinase-3 (MMP-3), thereby reducing inflammation. These results indicate the potential of cordycepin as a neuroprotective agent against ischemic brain injury [114]. In a rat model of cerebral ischemia–reperfusion (IR), *C. sinensis* mycelium (CSM) demonstrated neuroprotective effects by significantly reducing inflammation. It inhibited NF-kappaB activation, decreased inflammatory markers (IL-1β, TNF-α, iNOS, ICAM-1, and COX-2), and prevented PMN cell infiltration, indicating its potential to protect against cerebral IR injury [115].

### 6.4. Arrhythmia

A large cohort study revealed that both short-term and chronic exposure to outdoor PM air pollutants is associated with a heightened risk of arrhythmia [116]. *Cordyceps* has gained attention for its cardiovascular effects, working through various mechanisms, such as the direct dilation of blood vessels or the activation of M-cholinergic receptors, leading to improved coronary and cerebral blood circulation. In addition, *Cordyceps* has the potential to treat cardiac arrhythmias by correcting abnormalities in rhythmic contractions, indicating its therapeutic value for cardiovascular health [117]. Thirteen trials involving 1164 participants studied the effects of Ningxinbao capsules plus routine drugs on tachyarrhythmia, comparing 586 participants in the experimental group with 578 participants in the control group who received only routine treatment [118,119,120]. All participants received standard care for their primary condition and symptom relief. Eleven of these trials reported total effectiveness rates, showing that the experimental group had a significantly higher effectiveness rate than the control group, although notable heterogeneity was observed across the studies. Five trials used the effectiveness rate as the primary outcome, and an analysis of these five trials (n = 461) demonstrated a significantly higher effectiveness rate in the Ningxinbao group than in the routine treatment group (RR = 1.24; 95% CI, 1.15–1.35; z = 5.42; *p* < 0.00001; I^2^ = 37%) [121]. *Cordyceps* has demonstrated a positive effect on arrhythmia treatment, mainly by regulating adrenergic signaling in cardiomyocytes and modulating the PI3K-Akt signaling pathway [119,122].

## 7. Conclusions

This review highlights the therapeutic potential of the *Cordyceps* genus in treating air pollutant-induced respiratory and vascular diseases. Compounds such as adenosine, cordycepin, nucleotides, polysaccharides, fatty acids, sterols, and cyclic peptides in *Cordyceps* exhibit anti-inflammatory, immunomodulatory, lung-protective, and antiviral effects and alleviate ischemic cardiovascular conditions, as demonstrated in preclinical and clinical studies. *Cordyceps* species are currently being investigated in clinical trials for their potential to improve lung function following air pollutant exposure, aid in COVID-19 treatment, and serve as supportive therapy for lung cancer, a condition that is often aggravated by air pollution. Additionally, *Cordyceps* demonstrates potential in addressing thrombosis, providing neuroprotection in cerebral ischemia–reperfusion injury, and managing arrhythmias, which may be exacerbated by air pollutants. Key findings include its fibrinolytic and antiplatelet effects in preventing thrombosis, the neuroprotective properties of cordycepin and *C. sinensis* mycelium (CSM) against ischemic injury, and the ability to modulate adrenergic signaling and the PI3K-Akt pathway in arrhythmia management. Further research is essential to isolate and validate these bioactive compounds through well-designed, high-quality clinical trials targeting diseases associated with air pollution.

## Figures and Tables

**Table 1:** Chemical compounds related to air pollutants of *Cordyceps* spp., and their mode of action for respiratory diseases.

Species	Compounds	Factor	Mode of Action	Reference
*C*. *jiangxiensis*	Jiangxienone		Anti-lung cancer	[29]
*C*. *guangdongensis*	Polysaccharide	Tobacco smoking	Anti-inflammatory activity on chronic bronchitis	[44]
*C*. *guangdongensis*		Influenza virus H9N2	Antivirus activity	[44]
*C*. *militaris*	Cordycepin	COVID-19	Polyadenylation inhibitor with therapeutic potential against COVID-19	[45]
*C. militaris*	Beta-sitosterol, cholest-5-en-3β-ol, 3β, and 7α-Dihydroxycholest-5-ene	COVID-19	Controlling the cytokine storm in COVID-19	[46]
*C. militaris*	Acidic polysaccharide (APS)	Influenza A virus	Anti-influenza effect	[47]
*C. militaris*	Cordycepin	Lipopolysaccharide (LPS)	Reduces LPS-induced acute lung injury through the reduction in oxidative stress and inflammation	[48]
*C. militaris*		Silica	Inhibits silica-induced pulmonary inflammation	[49]
*C. militaris*	Cordycepin		Induces apoptosis in human lung cancer cells	[50]
*C. militaris*	Cordycepin		Triggers apoptosis by Caveolin-1-mediated regulation of JNK and Foxo3a in human lung adenocarcinoma	[51]
*C. militaris*	miR-1321 and miR-3188	Cigarette smoke extract	Alleviates the severity of murine acute lung injury	[52]
*C. ophioglossoides*	Cordycepol A	Cigarette smoke extract	Anticancer activities	[53]
*C. ophioglossoides*	Alkali-soluble polysaccharide		Antitumor	[33]
*C. sinensis*	Cordycepin, adenosine	Bleomycin	Antifibrogenic properties	[54]
*C. sinensis*	Exopolysaccharide		Immunocyte activity of tumor-bearing mice	[55]
*C. sinensis*	Ergosterol	Cigarette smoke extract	Inhibits cigarette smoke extract-induced COPD	[56]
*C. sinensis*		Cigarette smoke extract	Inhibits cigarette smoke extract-induced senescence in bronchial epithelial cells	[57]
*C. sinensis*	Cordycepin		Antitumor effect of cordycepin (3′-Deoxyadenosine) on murine lung carcinoma cells through adenosine A3 receptor stimulation	[58]
*C. sinensis*	Cordymin		Anti-inflammatory	[16]
*C. sinensis*	5,6-Epoxy-24(r)-methylcholesta-7,22-dien-3β-ol		Antitumor sterols from the mycelia of *C*. *sinensis*	[59]
*C. sinensis*	Cordyceps acid		Alleviates lung cancer in nude mice	[60]
*C. sinensis*			A protective effect against LPS-induced ALI in mice	[61]
*C. ciacadae*	Ergosterol peroxide		Inhibits phytohemagglutinin-induced T-cell proliferation	
*C. sphecocephala*		Ovalbumin	Anti-asthmatic activities	[62]

**Table 2:** Ongoing clinical trials on the use of *Cordyceps* spp.

*Cordyceps* Species	Identifier	Study Design	Effect	Clinical Improvement	Dose	Reference
*C. militaris*	NCT06138444	Randomized, double-blind, placebo-controlled clinical trial (20 healthy adults (10 males and 10 females), aged 20–60 years)	Natural immunostimulatory supplement	Significant increase in NK cell activity in males at 4 weeks (*p* = 0.049); significant increase in females at 8 weeks compared to placebo (*p* = 0.023); significant reduction in IL-1β levels in males at 8 weeks (*p* = 0.049); significant reduction in IL-6 levels in females at 8 weeks (*p* = 0.047)	75 mL of FCM containing 2.85 mg of cordycepin or placebo/day for 60 days	[23]
*C. militaris*	IRB No. 2013-02-009	Randomized, double-blind, placebo-controlled clinical trial (100 healthy adults aged 20–70 years with a history of at least two colds in the previous year)	Enhanced innate immune function; improved mucosal immunity relevant to respiratory defense; no adverse effects or abnormalities in liver, kidney, or hematological markers	A statistically significant increase in NK cell cytotoxicity was observed in the Cordyceps group compared to placebo (*p* = 0.047); a significant elevation in serum IgA levels (*p* = 0.035); safe and well-tolerated over 12 weeks of use	375 mg of CM ethanolic extract of 2 tablets/twice/day for 12 weeks	[76]
*C. militaris*		Open-label clinical trial (62 male patients diagnosed with benign prostatic hyperplasia (BPH))	Increasing urinary flow, decreasing the size of the prostatic gland, and alleviating micturition symptoms	Significant increase in urinary flow (*p* = 0.025); significant decrease in prostate volume (*p* = 0.016)	250 mg of 2 *Cordyceps militaris* fruiting bodies capsules, twice daily after meals for 12 weeks	[95]
*C. militaris*		Single-center, randomized, double-blind, placebo-controlled clinical trial (57 Korean adults aged 20–65 years with mild liver dysfunction (ALT 1.5–3× upper limit of normal))	Protect against the progression of fatty liver or cirrhosis in patients with mild liver dysfunction	Liver computed tomography (CT) Hounsfield units (HU): Mean increase of 21.43% ± 45.11% in the *Cordyceps* group vs. 9.64% ± 11.41% in the placebo group after 8 weeks; not statistically significant (*p* = 0.0987); no adverse effects or abnormal laboratory findings reported	1.5 g/day of *C. militaris* (2 capsules/doses, twice per day) for 4 weeks	[96]
*C. sinensis*	NCT06054438	Randomized, waitlist-controlled clinical trial (110 long COVID patients (55 per group)	*Cordyceps sinensis* mycelium extract (Cs4) significantly improved fatigue, sleep quality, respiratory symptoms, and overall quality of life in long COVID patients over 12 weeks	Significant improvement in the Cs4 group compared to control (MD: −10.1; 95% CI: −14.1 to −6.1; *p* < 0.001); significant reduction in fatigue (MD: −8.1; 95% CI: −14.2 to −2.0; *p* = 0.011); significant improvement in sleep quality (MD: −2.9; 95% CI: −4.6 to −1.2; *p* = 0.001); significant improvement in respiratory symptoms (MD: −6.3; 95% CI: −11.4 to −1.2; *p* = 0.018)	One Cs4 capsule (each 400 mg) 4 times/day for 12 weeks	[97]
*C. sinensis*		Randomized, double-blind, placebo-controlled clinical trial (37 healthy elderly Chinese volunteers)	Cs-4 could improve oxygen uptake or aerobic capacity and ventilation function and resistance to fatigue among elderly people in exercise.	Increased maximum oxygen uptake (VO_2_max) from 1.88 ± 0.13 to 2.00 ± 0.14 L/min in the Cs-4 group (*p* = 0.050); no significant change in the placebo group and anaerobic threshold	Cs-4 (3 g/day) for 6 weeks	[98]
*C. sinensis*		Randomized controlled trial (58 patients with ventricular arrhythmia)	Cs-4 (*Cordyceps sinensis*) improves exercise performance and might contribute to wellness in healthy older subjects; improvement in the treatment of ventricular arrhythmia, as evidenced by higher total effective rates and better electrocardiogram (ECG) outcomes	Observation group showed a higher total effective rate compared to the control group (*p* < 0.05); post-treatment ECG results in the observation group significantly improved compared to those in the control group (*p* < 0.05); no significant difference in the incidence of adverse reactions between the two groups (*p* > 0.05)	Cs-4 333 mg or placebo capsules 3 times a day for 12 weeks	[18]
*C. sinensis*		Systematic review and meta-analysis of randomized controlled trials (1238 patients with stable COPD (GOLD stages 2–3)	Meta-analysis showed that both CS preparations and CS formulae had potential benefits for lung function, exercise endurance, life quality, and improvement of symptoms for chronic obstructive pulmonary disease (COPD) of Global Initiative for Chronic Obstructive Lung Disease (GOLD) stages 2–3.	Quality of life: CS preparations led to a reduction in SGRQ scores by 4.57 points (95% CI: −7.53 to −1.61), indicating improved health-related quality of life. Symptom improvement: Patients receiving CS reported better symptom relief, with an odds ratio (OR) of 2.62 (95% CI: 1.71 to 4.03) for effective treatment compared to controls		[64]
*C. sinensis*	ChiCTR1900025707	Randomized, double-blind, placebo-controlled, multicenter clinical trial (240 patients diagnosed with chronic bronchitis [CB])	Reduced the frequency of acute exacerbations and improved key respiratory symptoms such as expectoration and wheezing	Patients receiving Bailing capsules reported significant improvements in expectoration (*p* = 0.012) and wheezing (*p* = 0.003) compared to the placebo group	Bailing capsule (Cs-C-Q80) 2.0 g, three times daily for 48 weeks	[14]
*C. sinensis*		Randomized controlled trial (60 patients with moderate persistent asthma)	The effect of the Dongchong Xiacao capsule on the airway inflammation in asthmatic patients	The treatment group exhibited a more significant reduction in serum levels of IL-4, sICAM-1, MMP-9, and IgE compared to the control group (*p* < 0.05 or *p* < 0.01)	Received standard therapy plus Dongchong Xiacao capsules for 2 months	[99]
*C. sinensis*		Randomized, double-blind, placebo-controlled, prospective trial (20 healthy elderly subjects aged 50–75 years)	Improves exercise performance among healthy older adults by improving metabolic and ventilatory thresholds	The Cs-4^®^ group exhibited a significant increase in metabolic threshold (above which lactate accumulates) by 10.5%, from 0.83 ± 0.06 to 0.93 ± 0.08 L/min (*p* < 0.02). An 8.5% increase in ventilatory threshold (above which unbuffered H^+^ stimulates ventilation) was observed, from 1.25 ± 0.11 to 1.36 ± 0.15 L/min (*p* < 0.05)	Cs-4 333 mg or placebo capsules 3 times a day for 12 weeks	[18]
*C. sinensis*	ChiCTR2100048419	Randomized controlled trial (72 patients with stable COPD)	Pulmonary rehabilitation training can enhance lung function, quality of life, and T cell immune function in stable-phase COPD patients. Perhaps the recovery of T-cell immune function is the root of the patient’s improvement	The experimental group showed significant improvements in FEV_1_% and FEV_1_/FVC% compared to the control group after 12 weeks (*p* = 0.002 and *p* = 0.009, respectively). Increases in CD3^+^% and CD4^+^% were significant in the experimental group compared to the control group (*p* = 0.037 and *p* = 0.046, respectively). The CD4^+^/CD8^+^ ratio improved significantly in the experimental group (*p* < 0.001)		[100]
*C. sinensis*	CRD42022333681	Systematic review and meta-analysis of randomized controlled trials (RCTs) (928 patients across 12 RCTs)	Adjuvant treatment with CS of lung cancer not only improves the tumor response rate, quality of life, and immune function but also reduces the incidence of ADRs and radiation pneumonitis	Tumor response rate increased (RR: 1.17; 95% CI: 1.05–1.29; *p* = 0.00); significant improvements in CD4 (MD: 4.98; 95% CI: 1.49–8.47; *p* = 0.01), CD8 (MD: 1.60; 95% CI: 0.40–2.81; *p* = 0.01), NK cells (MD: 4.17; 95% CI: 2.26–6.08; *p* = 0.00), IgA (MD: 1.29; 95% CI: 0.35–2.24; *p* = 0.01), IgG (MD: 3.95; 95% CI: 0.98–6.92; *p* = 0.01), and IgM (MD: 6.44; 95% CI: 0.63–12.26; *p* = 0.03)	6 g/day and 21 days/3–4 cycles	[15]
*Paecilomyces hepiali*	NCT 02814617	Randomized, double-blind, placebo-controlled clinical trial (79 healthy adults (39 in the CBG-CS-2 group, 40 in the placebo group))	Enhancing cell-mediated immunity in healthy adults	Significant increase of 38.8 ± 17.6% in NK cell activity from baseline in the CBG-CS-2 group compared to the placebo group after 8 weeks (*p* < 0.019)	Two CBG-CS-2 capsules (twice per day) or two placebo capsules (twice per day) per day (1.68 g/day) after breakfast and dinner for 8 weeks	[101]

Information on clinical trial identifiers (NCT and CRD) is based on records registered at ClinicalTrials.gov and the Chinese Clinical Trial Registry.

## Abbreviations

The following abbreviations are used in this manuscript:

MDPI	Multidisciplinary Digital Publishing Institute
DOAJ	Directory of Open Access Journals
TLA	Three-letter acronym
LD	Linear dichroism
